# New Medical Journals

**Published:** 1868-03

**Authors:** 


### New Medical Journals.

"Half-Yearly Compendium of Medical Sciences."—This journal is designed to embrace a digest of the whole field of medical literature with a view to that conciseness and practicability which best suits the general practitioner. Edited by Drs. S. W. Butler and D. G. Brinton, Philadelphia. Price $3 per annum, in advance.

"New Orleans Journal of Medicine."—This medical periodical is a consolidation of the two medical journals, lately published in New Orleans. It is to be published quarterly, and to be under the editorial charge of Dr. W. S. Mitchell, M. D. and W. H. Lewis, M. D. Price $6 in advance.

"L'Evénement Médical."—This weekly journal is under the editorship of Prof. Piorry, France. The numbers are regularly received by us in exchange. Prof. Piorry has recently been elected member of the Academy of Medicine.

"The Medical Repertory."—The first number of this journal has been received Each number is to contain thirty-two pages. It is designed to make this journal the exponent of the medical profession of the West. Prof. J. A. Thacker has the editorial management.

